# Mosquito-borne viruses circulating in Kinshasa, Democratic Republic of the Congo

**DOI:** 10.1016/j.ijid.2017.01.016

**Published:** 2017-04

**Authors:** Kennedy Makola Mbanzulu, Roger Wumba, Jean-Pierre Kambala Mukendi, Josué Kikana Zanga, Fortunate Shija, Thierry Lengu Bobanga, Michel Ntetani Aloni, Gerald Misinzo

**Affiliations:** a Department of Tropical Medicine, Infectious and Parasitic Diseases, Faculty of Medicine, University of Kinshasa, PO Box 747 Kinshasa XI, Kinshasa, Democratic Republic of the Congo; b Department of Microbiology, Parasitology and Immunology, College of Veterinary and Medical Sciences, Sokoine University of Agriculture, Morogoro, Tanzania; c Department of Paediatrics, Faculty of Medicine, University of Kinshasa, Kinshasa, Democratic Republic of the Congo

**Keywords:** Mosquitoes, Mosquito-borne viruses, Arboviruses, Kinshasa, Democratic Republic of the Congo

## Abstract

**Background:**

Diseases caused by mosquito-borne viruses are among the most important emerging diseases that threaten human and animal health, particularly in Africa. However, little attention has been paid to these diseases in the Democratic Republic of the Congo (DRC). The present cross-sectional study was undertaken between March and May 2014 to investigate the presence of mosquito-borne viruses in mosquitoes collected from five municipalities of Kinshasa, DRC.

**Methods:**

Mosquitoes were collected using BG-Sentinel traps and battery-powered aspirators. Female mosquitoes were pooled according to their genera and sampling locations, preserved in RNAlater, and later screened for viruses using reverse transcription PCR (RT-PCR) assays.

**Results:**

A total of 2922 mosquitoes were collected and 29 pools of female mosquitoes, containing approximately 30 mosquitoes each, were tested. Twelve of the 29 (41.4%) mosquito pools were found to be infected with at least one arbovirus, with eight (27.5%) pools positive for *Alphavirus*, nine (31%) for *Flavivirus*, and five (17.2%) for *Bunyaviridae*. Chikungunya, o’nyong’nyong, and Rift valley fever viruses were detected.

**Conclusions:**

The present study shows that mosquitoes in Kinshasa carry mosquito-borne viruses that may have serious public health implications. Further investigations on the presence of mosquito-borne viruses in the human and livestock populations of Kinshasa and DRC are recommended.

## Highlights

• Adult mosquitoes collected in Kinshasa, the Democratic Republic of the Congo (DRC), were screened for viruses in this study.
• Approximately 40% of mosquitoes were found to be infected with *Alphavirus*, *Flavivirus*, and/or *Bunyaviridae*.
• Chikungunya, o’nyong’nyong, and Rift valley fever viruses were found in mosquitoes.
• Habitats favouring mosquito breeding and viral transmission were found in Kinshasa.
• Screening for mosquito-borne viruses in humans and livestock in DRC is recommended.

## Introduction

Arboviral diseases are among the most important emerging infectious diseases threatening public health in many countries of the world.^1^ The Democratic Republic of the Congo (DRC) is the second largest African country and shares a long boundary with nine countries, including Congo, Central African Republic (CAR), South Sudan, Uganda, Rwanda, Burundi, Tanzania, Zambia, and Angola. Over the past decade, significant population movements arising from conflicts may have contributed to the introduction of arboviral diseases into new areas. Indeed, 2.4 million people were displaced within DRC because of war and 46 300 refugees have come in from neighbouring and endemic countries.^2^ Because of its large population of non-human primates and other animal reservoir hosts, DRC is believed to be the origin of several important emerging viruses of humans.^3^ However, few studies have been conducted on arboviruses within the country, hampering a reliable estimation of the current status and burden of arboviruses in DRC.

A recent study in the Congo basin that assessed the role of wildlife species as reservoirs for arboviruses (flaviviruses and alphaviruses) by testing sera from various animals, such as buffaloes, elephants, duikers, mandrills, gorillas, monkeys, and chimpanzees, showed the presence of antibodies against chikungunya, o’nyong’nyong, West Nile, dengue, and yellow fever viruses.^4^ In 1999 and 2000, two outbreaks of febrile illness were reported in humans following heavy rains in Matete and Kingabwa townships of the DRC capital city, Kinshasa. An estimated 50 000 humans cases were reported, and chikungunya virus was identified as the cause of these febrile illness outbreaks.^5^ A high proportion of tested sera from these cases presenting with febrile illness were found to have immunoglobulin M (IgM) antibodies against chikungunya virus but not against dengue virus, West Nile virus, or bunyaviruses.^5^ Later on, when the chikungunya viruses were isolated and their partial genomic sequences determined, the isolates from DRC were found to constitute a homogeneous group that was more closely related to CAR and Ugandan isolates than to Tanzanian and South African strains.^6^ These data called for urgency in conducting and expanding disease surveillance in DRC for emerging viruses that may represent an imminent threat to the population of Africa. However, disease prevention efforts have mostly focused on malaria and little has been done to understand the burden of arboviral disease and to mitigate the risks of possible large-scale outbreaks occurring in DRC.

The present study was conducted to investigate the presence of arboviruses such as yellow fever virus, dengue virus, o’nyong’nyong virus, Rift Valley fever virus, and chikungunya virus, in order to provide information that could serve as an early warning for possible outbreaks and to understand the exposure risk in certain selected areas of Kinshasa. The findings of this study may assist in the development and implementation of strategies for the control of arboviral diseases.

## Methods

### Study area

**Figure 1:**
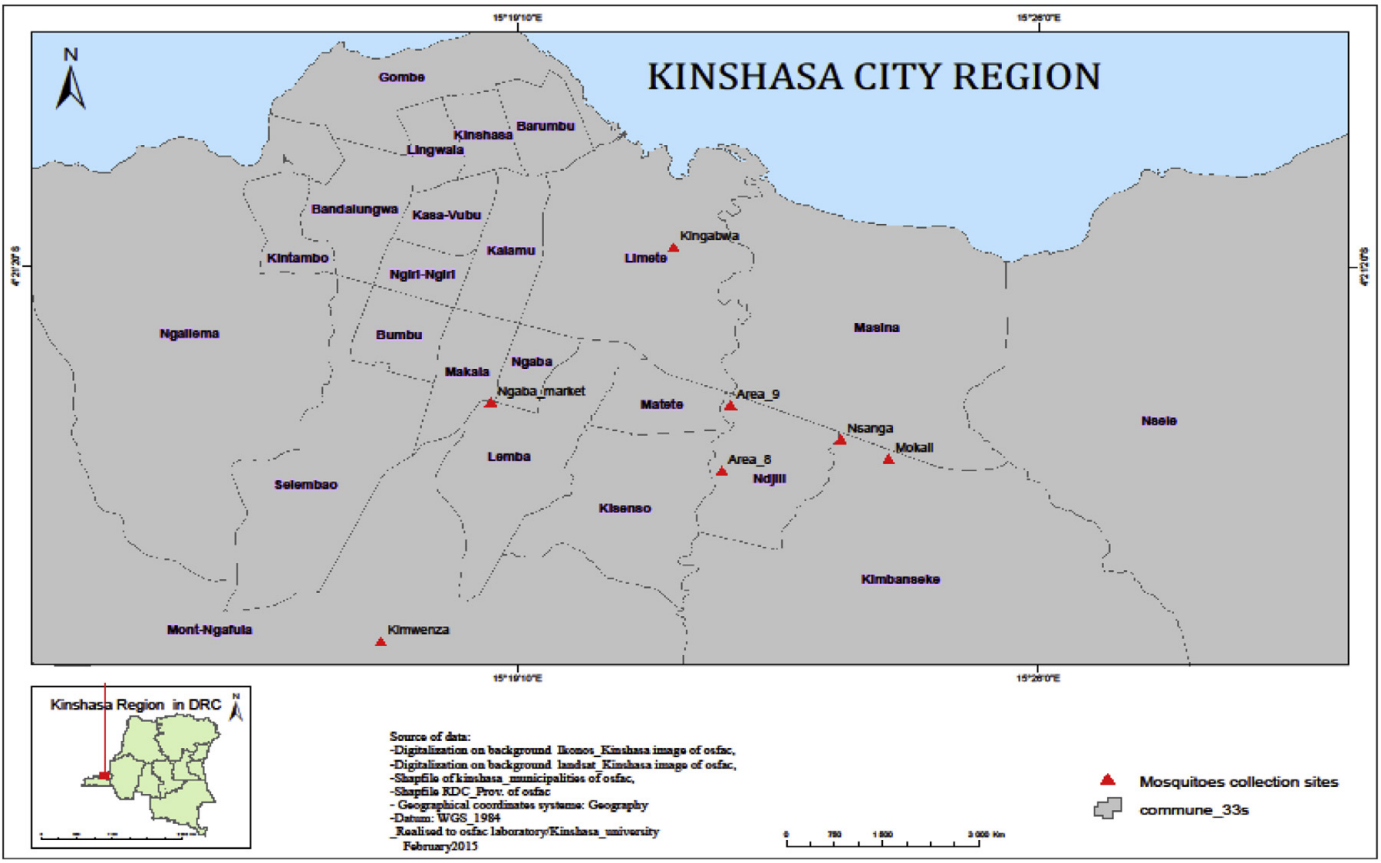
Map of Kinshasa showing the mosquito sampling locations. Adult mosquitoes were collected from Kimwenza (Mount Ngafula municipality), Kingabwa (Limete municipality), Area 8 and 9 (Ndjili municipality), Nsanga and Mokali (Kimbanseke municipality), and Ngaba market (Ngaba municipality). The insert shows the location of Kinshasa City in the Democratic Republic of the Congo.

Mosquitoes were collected in selected areas within Kinshasa (Figure 1), the capital city of DRC, located at 4° 19′ 30′′ S and 15° 19′ 20″ E. Kinshasa covers an area of 9965 km^2^, of which 90% is rural. It borders Brazzaville across the wide Congo River and is surrounded by four provinces, including Kongo-Central in the south and Kwilu, Kwango, and Mai-Ndombe in the north and in the east. The climate of Kinshasa is characterized by two seasons: a dry season from the second half of May to September and a rainy season from October to the first half of May, with a short break in February. Kinshasa experiences an average of 1482 mm of rainfall per year and has an average annual temperature of 25.2 °C and average annual relative humidity of 80.3%. The landscape ranges from the larger plain to some hills on the periphery, and a considerable hydrographic network crosses Kinshasa. The soils are sandy, sandy-argillaceous to argillaceous, with vegetation including steppes, semi deciduous and riverine forest islands, and wooded and grassy savannah. Kinshasa is divided into 24 municipalities.

The sampling sites for the present study are known for being the most malaria-endemic areas. These study sites were chosen because they possess favourable mosquito breeding habitats and a history of occurrence of arboviral disease outbreaks. The five study sites selected included Kimwenza, Kingabwa, Ndjili, Kimbanseke, and Ngaba (Figure 1). Kimwenza is a semi-rural area of Mont-Ngafula municipality set on a plateau in western Kinshasa, where a bonobo sanctuary is located and where the last epidemic of chikungunya occurred. Kingabwa is a suburban area of Limete municipality in the north-eastern part of Kinshasa along the Congo River, where rice agriculture is practiced and previous urban chikungunya outbreaks have occurred. Ndjili municipality is located in south-eastern Kinshasa along the Ndjili River, where pig farming, agriculture, and automobile repairs are conducted. Kimbanseke municipality is the most populated suburban area located in the eastern part of Kinshasa and is where vegetable cultivation is practiced and the swampy areas of Mokali and Nsanga are located. Ngaba municipality is an area surrounding the main market in central Kinshasa, characterized by a polluted environment due to the lack of an appropriate drainage sewage system and waste collection.

### Mosquito collection

The present cross-sectional study was conducted between March and May 2014 in order to detect the presence of mosquito-borne viruses in mosquitoes. Adult mosquitoes were collected using unbaited BG-Sentinel traps (Biogents AG, Regensburg, Germany) that were set at 05:00 h and collected 24 h later. Daytime collections were made in order to collect day-biting Aedes mosquitoes. Resting mosquitoes were collected using hand-held battery-powered aspirators. Collections were made over a minimum of 3 days at each site. Collected mosquitoes were killed using alcohol vapour and identified to the genus level with the aid of morphological keys.^7^, ^8^, ^9^ Female mosquitoes were sorted into pools according to their genera and location of collection, and preserved in RNAlater (Ambion, Austin, TX, USA). Each pool contained a maximum of 30 mosquitoes that were afterwards stored at −20 °C before being shipped to Sokoine University of Agriculture, Morogoro, Tanzania for the molecular detection of mosquito-borne viruses.

### Screening for mosquito-borne viruses in mosquitoes

#### RNA extraction

After grinding each of the pools containing 30 female mosquitoes preserved in 1.5 ml of RNAlater, RNA was extracted using the QIAamp Viral RNA Mini Kit for nucleic acid extraction (Qiagen, Hilden, Germany); this was done in accordance with the manufacturer’s instructions. Briefly, 560 μl of lysis buffer containing carrier RNA and 140 μl of homogenized mosquito tissue supernatant were added to a 1.5-ml microcentrifuge tube. The contents were pulse-vortexed for 15 s and afterwards incubated at room temperature for 10 min to ensure complete viral particle and cellular lysis. Protein precipitation was conducted by adding 560 μl of absolute ethanol followed by pulse-vortexing for 15 s. Precipitates were settled by centrifugation at 13 000 *g* for 5 min. The supernatant was carefully withdrawn and passed through a silica-gel column, followed by washing of the column twice with 500 μl of each of the washing buffers AW1 and AW2, respectively. Finally, RNA was eluted by addition of 60 μl of buffer AVE equilibrated at room temperature. The viral RNA extracted was immediately stored at −80 °C until cDNA synthesis and amplification.

#### Arbovirus detection by reverse transcription PCR

Virological detection of mosquito-borne viruses in mosquito pools was conducted using reverse transcription PCR (RT-PCR). To convert extracted RNA into cDNA, a total volume of 20 μl containing 4 μl of RNA template added into 16 μl of a master mix containing 4 μl of 5 × VILO reaction mix, 2 μl of 10 × Superscript reverse transcriptase enzyme mix (Invitrogen, Carlsbad, CA, USA), and 10 μl nuclease-free water was used. After mixing by vortexing for 15 s, the tube contents were incubated in a GeneAmp 9700 PCR System (Applied Biosystems, Foster City, CA, USA) at 25 °C for 10 min, 42 °C for 60 min to achieve reverse transcription, followed by enzyme denaturation at 85 °C for 5 min and a hold at 4 °C.

The cDNA was used for PCR amplification using primers targeting *Flavivirus*, *Alphavirus*, and *Bunyaviridae*, as reported previously by Ochieng et al.^10^ Briefly, a total of 20 μl of master mix was prepared, containing 10 μl of 2 × Dream Taq PCR Master Mix containing DNA polymerase (Thermo Scientific, Carlsbad, CA, USA), 1 μl of each forward and reverse primers, 1 μl of cDNA, and 7 μl of nuclease-free water. The PCR cycling conditions included an initial denaturation step at 94 °C for 15 min, followed by 35 cycles each consisting of a denaturation step at 94 °C for 30 s, an annealing step at 57 °C for 60 s, and an extension step at 72 °C for 30 s, followed by a final extension step at 72 °C for 10 min. Samples that tested strongly positive during screening for *Flavivirus*, *Alphavirus*, or *Bunyaviridae* were further screened for specific viruses using species-specific primers, as reported previously by Ochieng et al.^10^ For the detection of specific viruses, PCR was performed in a total reaction volume of 25 μl: 12.5 μl of 2 × Dream Taq PCR Master Mix containing DNA polymerase (Thermo Scientific, Carlsbad, USA), 0.5 μl of each forward and reverse primers, 2 μl of cDNA, and 9.5 μl of nuclease-free water.

#### Visualization of RT-PCR products

The RT-PCR products were separated by electrophoresis in a 1.5% agarose gel in 0.5 × Tris–borate–ethylenediaminetetraacetic acid (TBE) buffer (SERVA, Heidelberg, Germany) stained with GelRed nucleic acid stain (Phenix Research Products, Candler, NC, USA). Each well was loaded with 5 μl of the PCR product and 1 μl of 6 × blue–orange DNA loading dye (Promega, Madison, USA). Samples were separated along with DNA ladder (Promega, Madison, USA) at 150 V for 30 min. The PCR products were visualized using a gel documentation system (EZ Gel Doc, Bio-Rad, France) and scoring was done based on the size of the PCR products.

## Results

### Number of mosquitoes collected

A total of 2922 mosquitoes were collected from the study sites, comprising 1986 *Aedes spp*, 631 *Culex spp*, 283 *Anopheles spp*, and 22 *Mansonia spp* (Table 1). The highest number of mosquitoes was collected from Kimwenza (*n* = 891), followed by Ndjili (*n* = 697), Kimbanseke (*n* = 528), Kingabwa (*n* = 413), and Ngaba (*n* = 393) (Table 1). *Aedes spp* were the most commonly collected mosquitoes at these sampling sites, except in Ngaba where *Culex spp* predominated (Table 1). Female mosquitoes were grouped into 29 pools based on location: Kimwenza (*n* = 9), Ndjili (*n* = 7), Kimbanseke (*n* = 5), Kingabwa (*n* = 4), and Ngaba (*n* = 4). These 29 pools of mosquitoes included 20 pools of *Aedes spp*, six pools of *Culex spp*, and three pools of *Anopheles spp*.

### Mosquito-borne virus detection in mosquitoes by RT-PCR

**Figure 2:**
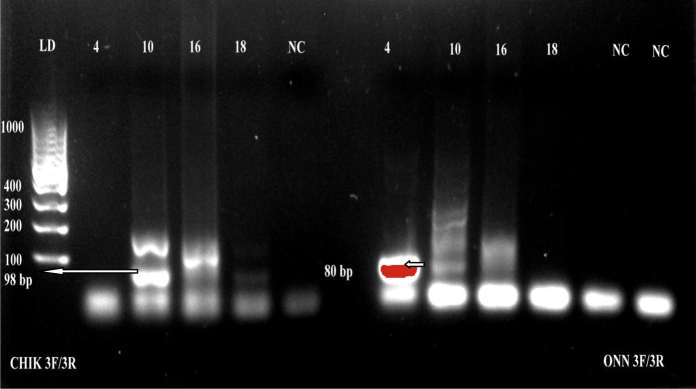
Detection of chikungunya and o’nyong’nyong viruses in female mosquito pools collected in Kinshasa. Amplification of chikungunya and o’nyong’nyong viruses using reverse transcription PCR (RT-PCR) targeting the 5′ non-translated region. The expected PCR product size for chikungunya was 98 base pairs, while it was 80 base pairs for o’nyong’nyong virus. NC: negative control; LD: ladder. Lane numbers indicate the pool identity.

Arboviruses were detected in 12 of the 29 pools (41.4%) and included *Alphavirus* (eight pools), *Flavivirus* (nine pools), and *Bunyaviridae* (five pools) (Table 2). Some pools showed mixed infection with two or three arboviruses. Amplification with specific primers for different members of each genus showed the presence of chikungunya virus, o’nyong’nyong virus (Figure 2), and Rift Valley fever virus in Aedes pools and o’nyong’nyong virus in the Anopheles pool. Positive pools were detected in all of the locations tested within Kinshasa (Table 1).

## Discussion

The abundance of mosquito vectors in an environment can favour the transmission of viruses. Furthermore, multiple feeding by mosquitoes can increase the probability of concurrent infection and viral genetic mixing.^11^ In the present study, the distribution patterns and abundance of mosquitoes were found to be restricted to certain areas, probably due to ecological and environmental adaptation. These findings are supported by the findings of an earlier study conducted by Karch et al., who found that the distribution of mosquito species in Kinshasa was non-homogeneous.^12^

Although the significant presence of *Aedes spp* reported in this study could be due to the use of BG-Sentinel traps, which are known to be more effective for catching day mosquitoes, the abundance of *Aedes spp* in some areas is likely linked to habitat and human activity.^13^ The presence of vegetable gardens and bamboo, and the inappropriate disposal of containers and used car tyres in Kimbanseke, Ndjili, and Kingabwa, as well as the tree-covered land of Kimwenza, constitute a favourable habitat for Aedes mosquito multiplication. A report from Pakistan demonstrated that the used car tyre trade had contributed to the re-emergence of *Aedes aegypti*, along with the emergence of diseases such as dengue.^14^

The lack of a sewage drainage system and the uncontrolled population growth in suburban areas of Kinshasa, as well as in its peri-urban areas, has increased the level of environmental pollution. This could explain the abundance of *Culex spp* in Ngaba, where there is no proper waste collection and disposal or sewage drainage system, increasing the level of pollution and providing a suitable breeding habitat. These observations corroborate the findings of other studies performed in Cameroon^15^ and Ivory Coast,^16^ which showed that the strong presence of *Culex quinquefasciatus* is associated with a polluted environment and that it can be considered as a biological marker of urbanization.

The present study provided an opportunity to report the presence of certain arboviruses in Kinshasa, of which several have been reported previously.^6^, ^7^ However, this appears to be the first study to report the presence of Rift valley fever virus in Kinshasa. In general, the presence of most of the arboviruses goes unnoticed in malaria-endemic regions, where febrile illnesses are frequently considered to be malaria because of the similarity in their clinical presentation and lack of appropriate laboratory diagnostic capacity for differential diagnosis.^17^, ^18^ The lack of appropriate diagnosis and medical care leads to high mortality and huge economic losses, resulting in worsening poverty in Africa. Arboviral infections can most effectively be controlled through the use of vaccines. However, this is limited by a lack of registered vaccines for the majority of circulating arboviruses in Africa, except for vaccines against yellow fever virus for humans and Rift valley fever virus for livestock.^10^

The high number of arbovirus-positive pools reported in this study (12 from a total of 29 pools), showing the arboviruses circulating in Kinshasa, is alarming. It is proposed that further work to screen for arboviruses in Kinshasa, especially in the human population, is performed. Surveillance programmes designed to monitor virus activity in vectors provide a system for mapping disease distribution and information needed not only to assess risk, but also to identify vector species for targeted control.^19^

The presence o’nyong’nyong and Rift valley fever viruses in mosquitoes circulating in Kinshasa may reflect a lack of previous arbovirus surveillance, but could also be a sign of the emergence of these arboviruses. The absence of dengue in screened mosquitoes could also be due to the small sample size of this study. However, it has been shown that *A. aegypti* originating from Central Africa are less competent in transmitting dengue virus compared to those circulating in East Africa and other locations of the world.^20^ Field studies from Central Africa conducted in Gabon and Cameroon have shown a lower susceptibility of *A. aegypti* to become infected with dengue virus and chikungunya virus than *Aedes albopictus*.^21^ This variability in vector competence between populations of *A. aegypti* from different geographical origins is intricately linked to the genetic heterogeneity of the species and could be influenced by environmental factors, human activity, insecticide treatment, and modalities of water supply storage.^13^ No pool was also positive for Yellow fever virus, while yellow fever outbreaks have recently been reported. This could be ascribed to the absence of outbreaks during the mosquito collection period or to the fact that mosquitoes could be feeding on uninfected people.

The present study findings also suggest that the environment is playing an important role in the occurrence of these arboviruses, as revealed by the frequency of positive pools in the areas surrounding the Ndjili River where pig farms are present and agriculture is practiced, as well as in Kimwenza where a bonobo sanctuary is located. This is supported by findings from Kenya, where the pastoral regions of Garissa and Marigat showed the highest frequency and diversity of arboviruses compared to other regions.^10^

In conclusion, mosquitoes from five selected areas of Kinshasa were collected and analyzed in this preliminary study, with the objective of detecting the presence of mosquito-borne viruses. Mosquito-borne viruses of public health importance are circulating within mosquitoes in Kinshasa. The health authorities in Kinshasa should conduct surveillance for arboviruses, to establish effective prevention measures. Healthcare providers must take account of arboviral diseases in the differential diagnosis of febrile illnesses. Future studies should sample more areas in the province of Kinshasa and other parts of DRC to detect areas at risk of arboviral disease.

## Conflict of interest

The authors declare that there are no competing interests.

## Author contributions

KMM designed the study, participated in the field sample collection, performed laboratory and data analyses, and prepared the manuscript for publication. RW participated to the study design and data analyses, and read and critically revised the manuscript for publication. JPKM participated to the field sample collection, performed laboratory analyses, and read and critically revised the manuscript for publication. JKZ participated in the field sample collection, performed the data analyses, and read and revised the manuscript for publication. FS performed laboratory analyses and read and revised the manuscript for publication. TLB supervised the field sample collections and read and critically revised the manuscript for publication. MNA read and critically revised the manuscript for publication. GM participated in designing field and laboratory studies, supervised the laboratory analyses, performed data analyses, and read and critically revised the manuscript for publication.

## Figures and Tables

**Table 1:** Different species of mosquitoes and their abundance at each of the sampling sites in Kinshasa, Democratic Republic of the Congo (DRC)

Genus	Kimwenza	Kingabwa	Kimbanseke	Ndjili	Ngaba	Total
*Aedes*	672	304	349	554	107	1986
*Culex*	118	80	98	77	258	631
*Anopheles*	97	23	71	64	28	283
*Mansonia*	4	6	10	2	0	22
Total	891	413	528	697	393	2922

**Table 2:** Mosquito-borne viruses screened using reverse transcription PCR (RT-PCR) in adult female mosquito pools collected from different locations within Kinshasa, Democratic Republic of the Congo^a^.

Location	Mosquito genus	*Flavivirus*	*Alphavirus*	*Bunyaviridae*	CHIKV	ONNV	YFV	RVFV	DENV
Kimbanseke	*Culex*	–	–	–					–
	*Aedes*	±	±	±					–
	*Aedes*	–	–	–					–
	*Anopheles*	–	+	+	–	+		–	–
	*Aedes*	±	±	–					–
Kingabwa	*Aedes*	–	–	–					–
	*Aedes*	–	–	–					–
	*Culex*	±	–	–					–
	*Aedes*	–	–	–					–
Ndjili	*Aedes*	+	+	+	+	–	–	+	–
	*Aedes*	–	–	–			–		–
	*Aedes*		–	–					–
	*Anopheles*	–	–	–					–
	*Culex*	–	–	–					–
	*Aedes*	–	–	–					–
	*Aedes*	+	+	+	+	–	–	+	–
Kimwenza	*Anopheles*	–	–	–					–
	*Aedes*	–	–	–					–
	*Aedes*	–	–	–					–
	*Culex*	–	–	–					–
	*Aedes*	–	–	–					–
	*Aedes*	–		–			–		–
	*Aedes*	–	–	–					–
	*Aedes*	±	±	±					–
	*Aedes*	±	±	–					–
Ngaba	*Aedes*	±	±	–					–
	*Aedes*	–	–	–					–
	*Culex*	–	–	–					–
	*Culex*	–	–	–					–
Control^b^	Dengue	+	–	–	–	–	–	–	+
	RNase-free water	–	–	–	–	–	–	–	–

CHIKV, chikungunya virus; ONNV, o’nyong’nyong virus; YFV, yellow fever virus; RVFV, Rift Valley fever virus; DENV, dengue virus.

a +, strongly positive reaction; −, negative reaction; ±, weakly positive reaction.

b Dengue was used as the positive control, while RNase-free water was used as the negative control.
